# Monitoring occurrence and persistence of *Listeria monocytogenes* in foods and food processing environments in the Republic of Ireland

**DOI:** 10.3389/fmicb.2014.00436

**Published:** 2014-08-20

**Authors:** Dara Leong, Avelino Alvarez-Ordóñez, Kieran Jordan

**Affiliations:** Food Safety Department, Teagasc Food Research CentreMoorepark, Fermoy, Cork, Ireland

**Keywords:** Listeria monocytogenes, ready-to-eat foods, persistence, food safety, food processing

## Abstract

Although rates of listeriosis are low in comparison to other foodborne pathogenic illness, listeriosis poses a significant risk to human health as the invasive form can have a mortality rate as high as 30%. Food processors, especially those who produce ready-to-eat (RTE) products, need to be vigilant against *Listeria monocytogenes*, the causative pathogen of listeriosis, and as such, the occurrence of *L. monocytogenes* in food and in the food processing environment needs to be carefully monitored. To examine the prevalence and patterns of contamination in food processing facilities in Ireland, 48 food processors submitted 8 samples every 2 months from March 2013 to March 2014 to be analyzed for *L. monocytogenes*. No positive samples were detected at 38% of the processing facilities tested. Isolates found at the remaining 62% of facilities were characterized by serotyping and Pulsed Field Gel Electrophoresis (PFGE). A general *L. monocytogenes* prevalence of 4.6% was seen in all samples analyzed with similar rates seen in food and environmental samples. Differences in prevalence were seen across different food processors, food sectors, sampling months etc. and PFGE analysis allowed for the examination of contamination patterns and for the identification of several persistent strains. Seven of the food processing facilities tested showed contamination with persistent strains and evidence of bacterial transfer from the processing environment to food (the same pulsotype found in both) was seen in four of the food processing facilities tested.

## Introduction

Mild listeriosis, an infection of the gastrointestinal tract by *Listeria monocytogenes*, generally presents itself with typical “food poisoning” symptoms including abdominal cramps, nausea and diarrhea. However, *L. monocytogenes* has the ability to cross the epithelial barrier of the intestinal tract to cause more serious infection throughout the body including bacteremia. It can also cross the blood-tissue barrier which allows the bacteria to infect organs such as the brain or uterus, where it can cause severe life-threatening infections such as meningitis, encephalitis, spontaneous abortion, or miscarriage. Although the incidence of human listeriosis is comparatively low, at up to 30% it has the third highest mortality rate of all food borne pathogens (EFSA, 2014) and immunocompromised individuals are particularly at risk (Vazquez-Boland et al., 2001; Cartwright et al., 2013). According to the most recent EU data, the mortality rate was 12.1% for the 1642 cases reported in the year 2012 (EFSA, 2014).

Food processors need to be vigilant against *L. monocytogenes* as the bacterium is ubiquitous in the environment, therefore contamination of the food processing environment is highly likely and cross-contamination of *L. monocytogenes* to foods is seen to be a major route of food contamination (Pérez-Rodríguez et al., 2008). *L. mono*cytogenes can survive for long periods of time in a seemingly inhospitable environment such as a food processing facility due, in part, to its ability to resist various stresses (Moorhead and Dykes, 2004; Zhang et al., 2011) and its ability to form biofilm (Latorre et al., 2010; Cruz and Fletcher, 2011). Consistent identification of specific *L. monocytogenes* strains in food processing facilities over many years has shown that strains can persist in food processing environments. For example, Holch et al. (2013) used genome sequencing to demonstrate the persistence of two separate strains over 6 years in two different fish processing facilities and Vongkamjan et al. (2013) used ribotyping to show the 11 year persistence of a *L. monocytogenes* strain in a smoked fish processing facility (Vongkamjan et al., 2013).

Ready-to-eat (RTE) foods are in a higher risk category than other foods as the heat step of cooking, which would kill any *L. monocytogenes* present, is missing in these foods (Luber et al., 2011; EFSA, 2013). *L. monocytogenes* can replicate even at refrigeration temperatures so it is of concern especially in products with a long shelf life. In recent years, foodborne listeriosis outbreaks have included several RTE products for example cheese (Choi et al., 2014; Rychli et al., 2014), cantaloupe (Mccollum et al., 2013), and cooked ham (Hachler et al., 2013). A European Union baseline study of *L. monocytogenes* in RTE foods has been conducted in 2010 and 2011 and prevalence rates found were of 2.07% in meat products, 0.47% in cheese products, and a more concerning rate of 10.4% in seafood products (EFSA, 2013). According to current European Commission (EC) regulations, RTE foods which cannot support the growth of *L. monocytogenes* must contain fewer than 100 CFU/g during their shelf-life while there is a zero tolerance policy in place for *L. monocytogenes* in RTE foods which can support its growth or which are intended for infant consumption or as a medicinal food (EC, 2005).

The aim of this study was to monitor the occurrence and persistence of *L. monocytogenes* in foods and food processing environments of 48 food processing facilities in the Republic of Ireland by regular sampling and characterization of isolates by serotyping and Pulsed Field Gel Electrophoresis (PFGE).

## Materials and methods

### *L. monocytogenes* monitoring programme

From March 2013 to March 2014, a total of 48 food processing facilities from various food sectors, i.e., dairy (18 facilities), meat (12 facilities), seafood (8 facilities), fresh-cut vegetable (6 facilities), miscellaneous (4 facilities), were analyzed bimonthly for the presence of *L. monocytogenes*. Of these food processing facilities, 43 process RTE food products. The selection of food processing facilities allowed coverage of major geographic areas of the Republic of Ireland. Sampling packs, which consisted of a polystyrene box (DS Smith, UK) containing six pre-moistened 3M sponge-stick swabs (Technopath, Ireland), a sterile liquid container (VWR, Ireland), two sterile bags (VWR, Ireland), two cable ties, and two ice packs, were sent to all participating food processing facilities.

Food business operators (FBOs) received detailed instructions which included information on how to take swab samples, which areas to sample, the type of food samples required and on the packaging and shipment of the samples to the laboratory. For swab samples, all FBOs were asked to take samples from three specific areas: a drain in the main processing hall, an area of floor (1 m^2^) and a storage shelf. Because of the variation in layout of the facilities, the area to swab for the remaining samples was freely chosen by the FBO from anywhere in the food processing environment, although cutting areas, brine (if relevant), walls, other drains and pooled water were suggested as optimum locations. For food samples, FBOs were instructed to send two food samples which were at the stage of being ready to be sent from the processing facility.

Every second month, FBOs took 6 environment samples and sent them to the laboratory at Moorepark by overnight courier along with 2 food samples. Thirty-seven FBOs were initially enrolled in the monitoring programme and 11 further FBOs later showed their interest in joining the collaborative network at different stages during the sampling year. On the other hand, 3 FBOs no longer wished to take part in the analysis or went out of business and several other companies missed one or various sample submissions throughout the year's sampling.

### Isolation of *L. monocytogenes* from food and environmental samples

Samples were analyzed for the presence of *L. monocytogenes* by the ISO 11290-1 method, except that only one agar was used. After the environment swabs arrived at the laboratory, 100 ml of half-Fraser broth (VWR, Ireland), was added to bags containing 3M stick-sponge swabs, after which they were incubated at 30°C for 24 h. Then, a 0.1 ml aliquot was transferred to 10 ml of full-Fraser broth, which was further incubated at 37°C for 48 h. In addition, a 0.02 ml aliquot of the 1st enrichment broth was plated onto Agar Listeria acc. to Ottavani & Agosti (ALOA) agar plates (Biomérieux, UK), which were incubated at 37°C for 48 h. After incubation, 2nd enrichment broths were streaked onto ALOA agar plates, which were again incubated at 37°C for 48 h. For liquid or food samples, 225 ml of half-Fraser broth was added to 25 ml or 25 g of randomly selected analytical units of the food samples. Samples were then homogenized in a stomacher (Colworth Stomacher 400) for 4 min, and incubated at 30°C for 24 h. Subsequently, analysis of samples was continued by following the same approach used for environmental samples. The food samples were analyzed after their “best before date” so that any sample positive for *L. monocytogenes* did not create food recall issues.

After incubation, ALOA agar plates were examined for typical *L. monocytogenes* colonies (blue-green colonies with halo), and, if present, two characteristic *L. monocytogenes* colonies for each positive enrichment were streaked first onto Brilliance *Listeria* Agar (BLA) plates (Fannin, Ireland), which were incubated at 37°C for 48 h, and then onto Brain Heart Infusion (BHI) agar plates, which were incubated at 37°C for 24 h. Cryoinstant tubes (VWR, Ireland) were prepared by resuspending the bacterial mass from BHI agar plates, and were kept at −20°C for bio-conservation.

### Molecular characterization of *L. monocytogenes* isolates

All stocked isolates were confirmed as *L. monocytogenes* by multiplex PCR as previously described (Ryu et al., 2013). Isolates were also serogrouped by multiplex PCR and serotyped by antisera testing (Denka Seiken UK Ltd, UK) as described previously (Fox et al., 2009). This allowed differentiation of all serotypes except 4b and 4e which cannot be differentiated with the currently available antisera.

PFGE analysis was performed with the restriction enzymes *Asc*I and *Apa*I, in two separate experiments, according to the International Standard PulseNet protocol (PulseNetUsa, 2009). Isolate similarity dendrograms were generated using Bionumerics version 5.10 software (Applied Maths, Belgium), by the un-weighted pair group method with arithmetic mean (UPGMA) with tolerance and optimization settings of 1%, as previously described (Fox et al., 2012).

## Results

### *L. monocytogenes* occurrence in foods and food processing environments

Overall, 2006 samples were analyzed for the presence of *L. monocytogenes*, which accounted for 1574 environmental samples and 432 food samples. In general, 4.6% prevalence of *L. monocytogenes* was observed with slightly higher incidences in food samples (5.3%) than in environmental samples (4.4%). Positive food samples were obtained from all the food sectors and included cheese, smoked salmon, apple juice, mushrooms, milk, sausages, pudding, gammon, stuffing, and chicken samples. Regarding environments, although the majority of positive environmental samples were sampled from non-food contact surfaces, 16.0% of positive environmental samples were from food contact surfaces.

**Figure 1:**
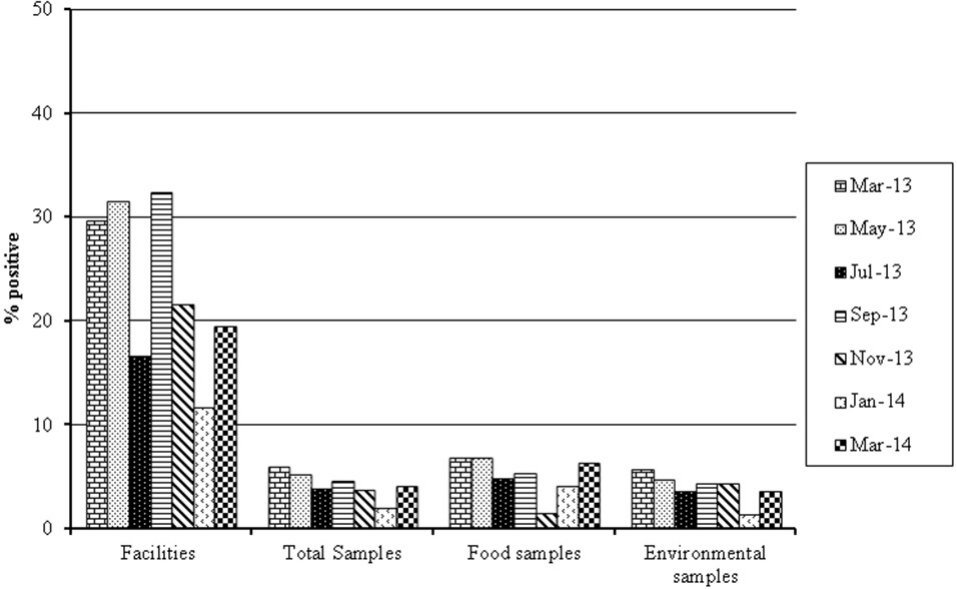
**Seasonal variation *L. monocytogenes* prevalence in samples from different processing facilities isolated between March 2013 and March 2014**.

In the total samples, slight variability in *L. monocytogenes* prevalence during the sampling year was observed. The lowest prevalences occurred in July 2013, November 2013, and January 2014 (3.9, 3.8, and 2.0%, respectively), while *L. monocytogenes* prevalence ranged between 4.2 and 6.0% for the rest of sampling months (Figure 1).

**Figure 2:**
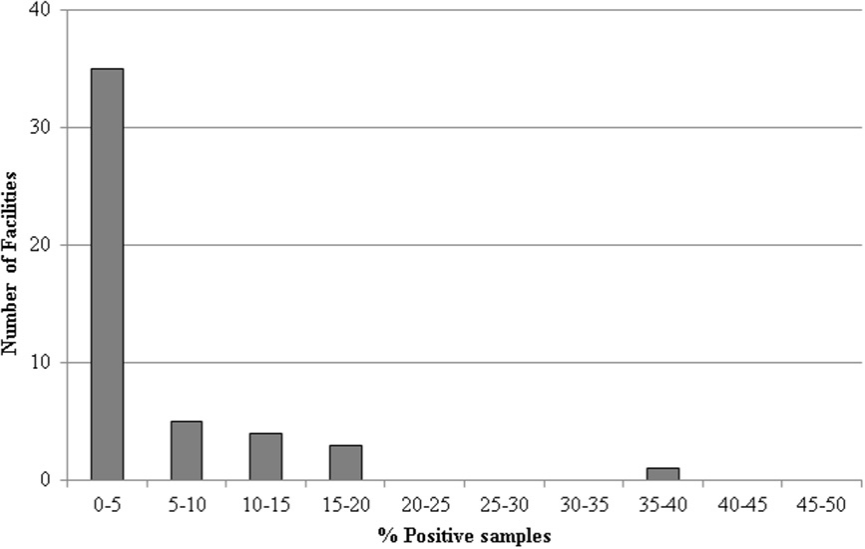
**Breakdown of the number of processing facilities positive for *L. monocytogenes* between March 2013 and March 2014**.

Thirty out of the 48 processing facilities analyzed had at least one positive sample over the course of the study. However, the majority of processing facilities consistently had a low prevalence of *L. monocytogenes*, in the range 0–5%, although some outliers occurred in which very high prevalence rates were found (Figure 2). Thus, 5, 4, 3, and 1 facilities presented prevalence rates of 5–10, 10–15, 15–20, and >30%, respectively (Figure 2).

Variability in *L. monocytogenes* prevalence rates was also observed among the different industry sectors. The highest rate of prevalence occurred in the vegetable sector in which 9.4% of samples tested positive for *L. monocytogenes*, followed by the meat sector at 4.2%, the dairy sector at 3.9% and the seafood sector at 1.6%. A miscellaneous group of processing facilities that process a range of foods belonging to different industrial sectors had a mean prevalence rate of 7.1%.

### Molecular characterization of *L. monocytogenes* isolates

When a food or environmental sample was found positive for *L. monocytogenes*, in 48.9% of cases both the first and second enrichment broths were positive, while in 20.4% of occasions the first enrichment was positive and the second was negative (most likely due to overgrowth by other related bacterial species) and in 30.7% of cases the first enrichment was negative but the second enrichment was positive (most likely due to a low contamination level of the sample). In any case, *two* colonies from each positive enrichment broth were isolated and further characterized by serotyping and PFGE analysis. Of the 370 isolates collected, the majority belonged to serotype 1/2a (41%), with 4b/4e (27%), 1/2b (17%), and 1/2c (15%) serotypes also being found but less frequently.

PFGE analysis of *L. monocytogenes* isolates provided information on strain diversity within positive samples. For the majority of samples (91.8%), indistinguishable PFGE pulsotypes were obtained for all strains isolated, while for 8.2% of samples isolates from more than one *L. monocytogenes* PFGE pulsotype were recovered, which suggests the possibility of food or environmental contamination with multiple *L. monocytogenes* strains.

PFGE analysis also allowed the identification of transfer of *L. monocytogenes* from environments to foods, or vice versa, and persistence within food processing facilities (Table 1). Indistinguishable pulsotypes in environmental samples and food samples of the same processing facility were observed for facilities 10, 22, 39, and 46. Facility 39 had an indistinguishable pulsotype in food and environmental samples at the same sampling time. Facilities 10, 22, and 46 had an indistinguishable *L. monocytogenes* pulsotype in food and environmental samples across different time points. Both facilities 22 and 46 had more than one type of food positive for *L. monocytogenes:* black pudding and sausages, and, chicken fillet and stuffing, respectively. In facilities 39 and 46, *L. monocytogenes* was isolated from food contact surfaces including a packing bench and shelf in facility 39 and a mincer and shelf in facility 46.

**Table 1:**
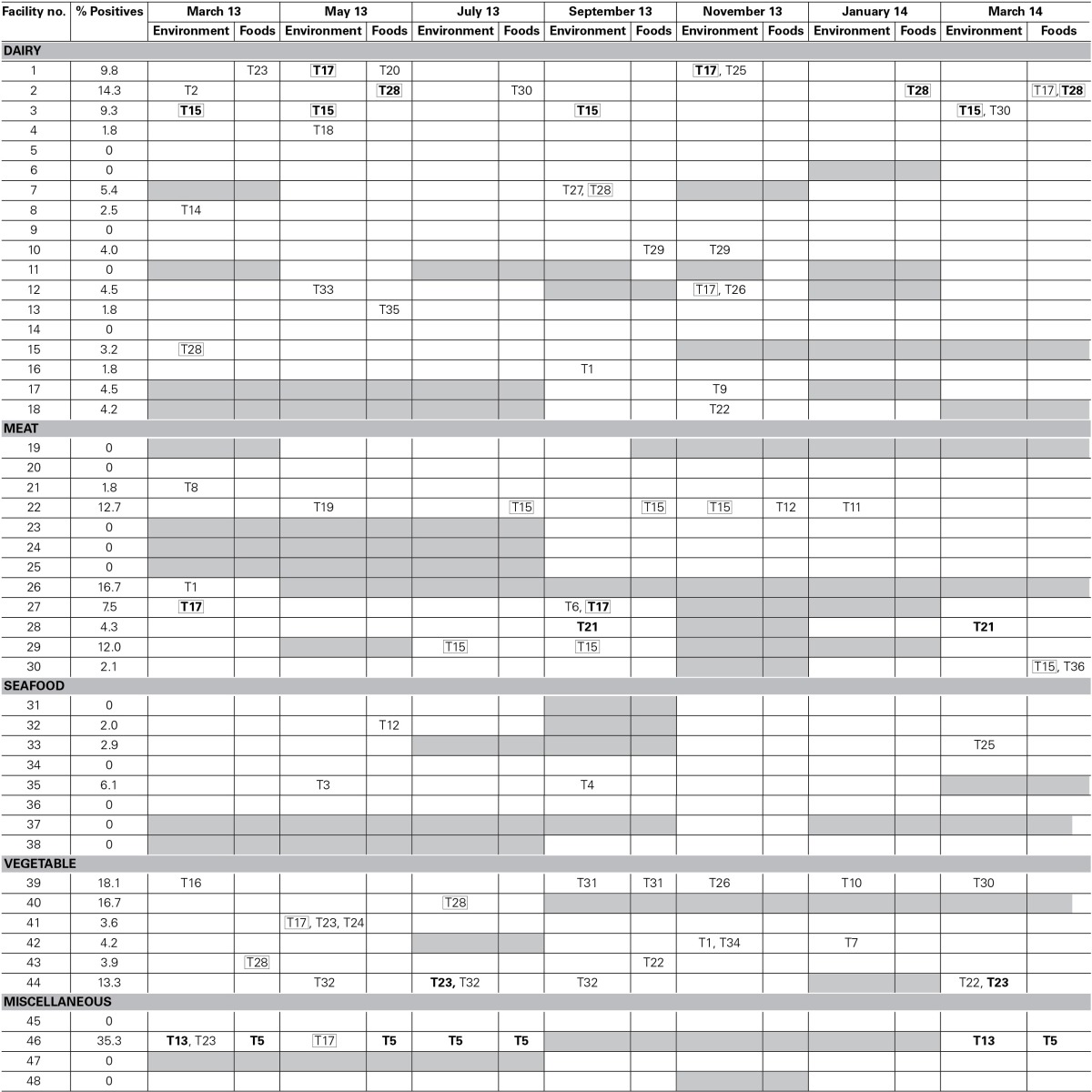
***L. monocytogenes* positives listed according to facility, sampling month, sample type, and pulsotype, e.g., T23**.

*L. monocytogenes* strains with indistinguishable PFGE profiles isolated at times 6 months or more apart are considered persistent strains for the purposes of the present study. Therefore, persistent strains occurred in seven separate facilities. Persistent strains were isolated from facilities of all industrial sectors except the seafood sector. In facility 44, a vegetable processor, pulsotype T32 (serotype 1/2b) was consistently isolated for over 6 months from drain and floor swabs and pulsotype T23 (serotype 4b/4e) was isolated for over 8 months from floor swabs. Similarly, at facility 46, a miscellaneous facility, pulsotype T13 (serotype 1/2a) was isolated separately up to a year apart from drain and floor swab samples and pulsotype T5 (serotype 1/2a) was consistently isolated for over 1 year from chicken fillet and stuffing food samples as well as from environmental swabs of drains.

**Figure 3:**
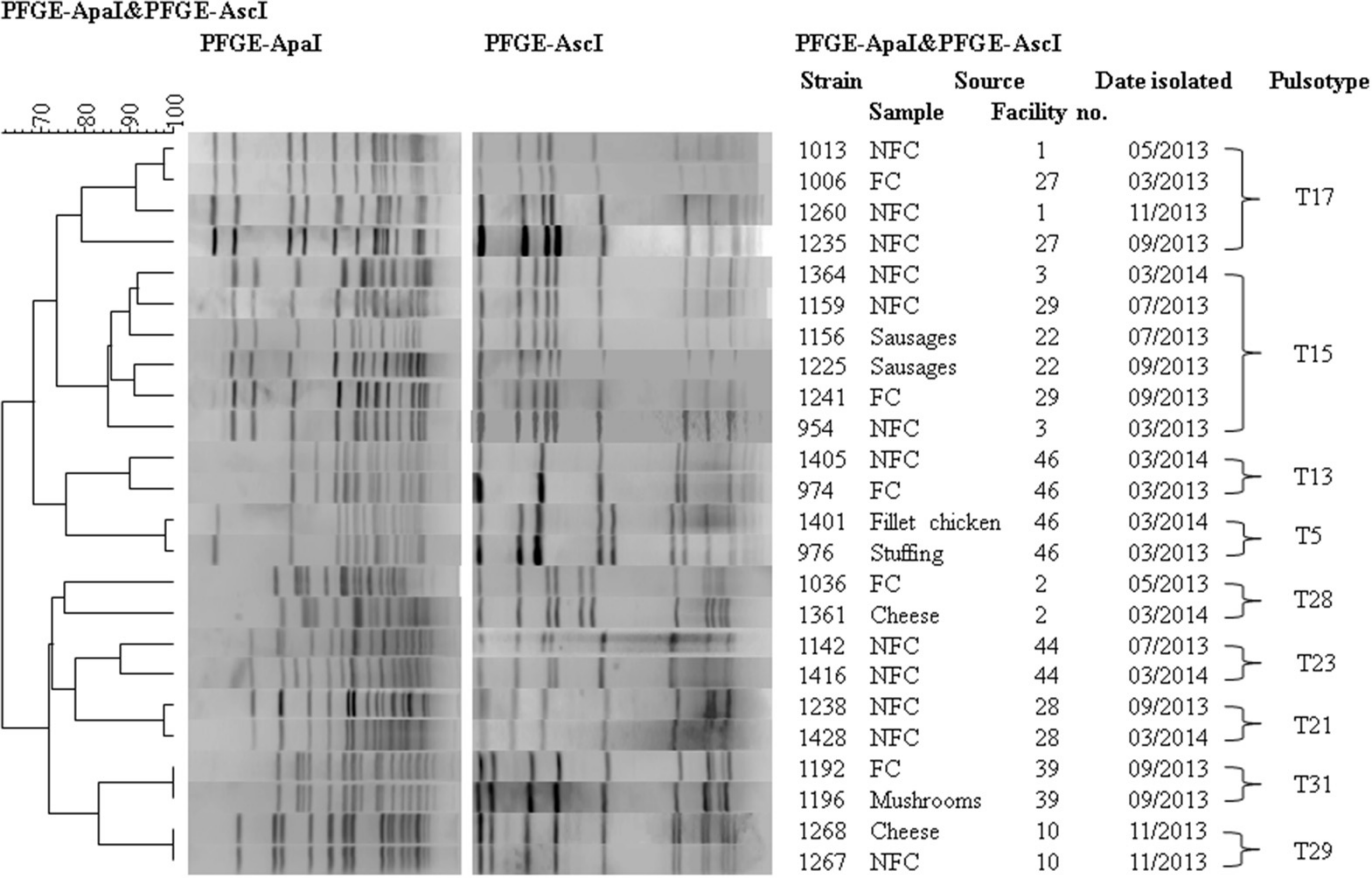
**Dendrogram of PFGE pulsotypes which persisted for over 6 months or were isolated from both food and environment samples within the same facility**. Examples of interest from each pulsotype are shown. FC, Food contact; NFC, Non-food contact.

A few persistent pulsotypes were found in more than one food business (Figure 3). This was the case for pulsotypes T15 (serotype 1/2c), T17 (serotype 1/2a), and T28 (serotype 4b/4e), which were isolated from several different facilities at various different sampling points during the sampling year (Figure 3). Pulsotype T15 was isolated in three separate facilities at several time points, pulsotype T17 in six separate facilities (in two of them it was isolated on more than one occasion) and T28 in five separate facilities (but only in one of them on more than one occasion). These persistent pulsotypes were not restricted to any particular industry sector with pulsotype T15 found in the dairy and meat sectors, pulsotype T17 in the dairy, meat, vegetable and miscellaneous sectors and pulsotype T28 in the dairy and vegetable sectors. All three of these pulsotypes were isolated from food samples at least once during the present study. Pulsotype T17 was isolated from cheese, pulsotype T15 was isolated from sausages and pulsotype T28 was isolated from milk, cheese and apple juice. Pulsotypes T17, T15, and T28 were all isolated from environmental swabs taken in both food contact and non-food contact areas.

Several other pulsotypes were isolated from more than one facility but were only found at one time point and, as such, have not yet been shown to be persistent.

## Discussion

### *L. monocytogenes* occurrence in food and food processing environments

Two thousand and six samples were submitted for analysis. Only 20 FBOs submitted all scheduled samples, therefore the number of samples submitted was less than originally planned. This was not detrimental to the study as a large number of samples were analyzed, allowing the examination of general *L. monocytogenes* prevalence trends across the different food sectors studied. The general *L. monocytogenes* prevalence of 4.6% agrees with the mean occurrence of *L. monocytogenes* in RTE foods across Europe found in the recent EFSA baseline study of approximately 4% (EFSA, 2013). Although the majority of facilities had consistently low or no presence of *L. monocytogenes*, outliers with high prevalence occurred in every sector except the seafood sector. The general, low *L. monocytogenes* prevalence was similar to that seen by Williams et al. (2011), who described an occurrence in small RTE food production facilities ranging from 1.7 to 10.8%. The higher prevalences of 23.68% in RTE seafood in Italy (Gambarin et al., 2012) and approximately 22% in various RTE foods in Spain (Sanchez et al., 2012) were closer to those of the outliers seen in the present study. Previous studies conducted in Ireland in farmhouse cheese processing facilities reported a higher prevalence of 13.1% (Fox et al., 2012) in the food processing environment. Significant contamination rates in food processing facilities greatly increase the chance of causing human listeriosis as was seen in Denmark in 2000 where human listeriosis cases were traced to a turkey processing facility in which *L. monocytogenes* prevalence ranged from 25.9 to 41.4% during production (Ojeniyi et al., 2000).

While both the dairy and meat sectors showed an average prevalence of 3.9 and 4.2%, respectively, previous studies have found a much higher prevalence in seafood samples (Garrido et al., 2009; Chen et al., 2010) than the present study. Chen et al. (2010) found 21.6% of samples positive from food and processing environment, Garrido et al. (2009) found 25% of food samples positive and in the current study, an occurrence of 1.6% was detected in food and the processing environment. The EFSA baseline study also showed a *L. monocytogenes* prevalence in smoked fish of 10.4%. The seafood industry is currently seen as a high risk industry for *L. monocytogenes* contamination e.g., an increase in listeriosis cases in Finland in 2010 was found to have been caused by two fishery plants which contained persistent *L. monocytogenes* strains (Nakari et al., 2014). It can be speculated that seafood processors in Ireland may be more aware of their vulnerability to *L. monocytogenes* contamination and have taken more steps to combat contamination in recent years which may have helped to reduce occurrence.

On the other hand, the food sector with the highest occurrence of *L. monocytogenes* was the fresh-cut vegetable sector, with a prevalence of 9.4%. The large variety of pulsotypes isolated from this sector indicates frequent contamination which did not originate at the same source. Therefore, a likely source of this contamination would be soil rather than materials from a common supplier, staff or equipment. To date, little research on *L. monocytogenes* in the vegetable processing industry has been conducted. The occurrence found in the present study is comparable to the *L. monocytogenes* occurrence found in mushrooms in Spain (Venturini et al., 2011). Only six facilities in the fresh-cut vegetable sector took part in the study, therefore it is not possible to determine if this is a general trend in the vegetable industry in Ireland. However, the data indicates that vegetable processors should be more vigilant against *L. monocytogenes*. As uncut vegetables are considered primary production, they are not included in the RTE category in regard to regulations concerning *L. monocytogenes* so are not subject to the same regulatory sampling as RTE foods. Therefore, vegetable processors may not be as aware of *L. monocytogenes* as other food processors from RTE sectors of the industry included in this study.

Regarding the miscellaneous sector, although a high occurrence of 7.1% was observed, only one of the four facilities (facility 46) tested positive for *L. monocytogenes*, with a very high contamination rate (35.3%).

For 37.5% of the facilities tested, *L. monocytogenes* was absent over the course of the present study. This requires further investigation to determine whether particular hygiene practices, equipment types, staff procedures etc. used in these facilities contributed to this absence of *L. monocytogenes*.

Differences in *L. monocytogenes* prevalence were not influenced by season. Not all facilities submitted samples on every occasion so it is possible that seasonal variation may have been observed if all of the samples had been submitted. However, this is unlikely as facility 46, which had the highest overall prevalence, still had a high prevalence of 25% in July 2013. In the literature, some debate exists concerning whether or not Listeria is subject to seasonal variation. Data which has found no seasonal variation (Ho et al., 2007; Esteban et al., 2009; Mohammed et al., 2010) exists alongside data which has found clear seasonal variation, including higher numbers of *Listeria* spp. occurring in both summer (Rivoal et al., 2010) and in winter months (Guerini et al., 2007).

### Molecular characterization of *L. monocytogenes* isolates

From the present study, the importance of analyzing isolates from both the 1st and 2nd enrichment can be seen. In only 48.9% of positive samples, both the 1st and 2nd enrichments were positive while in 51.1% of positive samples, either the 1st or the 2nd enrichment only was positive. In some studies, only the 2nd enrichment is plated for *L. monocytogenes* isolation; therefore, from the present data, that method would have missed 20.4% of positive samples. In addition, 8.2% of samples showed some strain diversity (multiple PFGE pulsotypes) indicating multiple colonization of the environment or food by more than one *L. monocytogenes* strain. This finding highlights the importance of isolating several strains from positive samples in monitoring programs in order to account for the possible strain diversity that can exist.

The ratio of serotypes found in the present study was in agreement with a 5 year surveillance report on similar food and food processing environment samples conducted in Italy in 2010 (Nucera et al., 2010) where serotype 1/2a isolates accounted for almost half of *L. monocytogenes* isolates followed by serotypes 4b/4e, 1/2b, and 1/2c in slightly varying lower percentages. Listeriosis outbreaks are most commonly caused by 4b/4e serotypes and to a lesser extent 1/2b serotypes while sporadic cases are commonly caused by 4b/4e, 1/2a, or 1/2c serotypes (Todd and Notermans, 2011). Therefore, all *L. monocytogenes* isolates found in the current study could have the potential to cause illness.

Several strains of *L. monocytogenes* were seen to persist over long periods of time in the food processing environment as their pulsotypes were identified repeatedly by PFGE. Long-term survival of strains in a food processing facility, such as these, confer a higher risk of bacterial transfer to food and therefore a higher risk of human exposure to the pathogen (Lambertz et al., 2013). Seven of the 48 facilities included in the study showed contamination with persistent strains. It is possible that at least some of these strains have adaptions which facilitate long-term survival in a food processing environment. These adaptations could include resistance to sanitizers/disinfectants, adaptation to cold or high salt conditions and ability to form biofilms (Holch et al., 2013). One opposing theory suggests that rather than strains possessing particular characteristics which contribute to their persistence, bacteria simply persist in harborage sites i.e., areas which cannot be effectively disinfected, and proliferate from these sites (Carpentier and Cerf, 2011).

Several pulsotypes were also seen to be common to several different facilities. There is no known epidemiological link between any of the facilities which shared *L. monocytogenes* pulsotypes. For example, T17 was isolated in facilities 1, 2, 12, 27, 41, and 46. These facilities belong to various industry sectors (dairy, meat, vegetable, and miscellaneous), are located throughout the country and are not known to share any suppliers, equipment, or staff. Further characterization, including ongoing genome sequencing, may shed light on whether or not certain strains have adapted to the food processing environment.

## Conclusion

The prevalence of *L. monocytogenes* in food and food processing environments among 48 FBOs in the Republic of Ireland was 4.6% and is equivalent to the prevalence rates found in RTE foods across the E.U. (EFSA, 2013). PFGE analysis allowed the identification of several strains as persistent in the food processing environment and several strains were also seen to occur in more than one facility. The general prevalence of approximately 5% of *L. monocytogenes* positives found in samples of both food and food processing environments, in addition to the evidence of bacterial transfer and persistence observed, emphasizes the need for food processors to be vigilant against *L. monocytogenes* contamination in order to avoid public health risks.

### Conflict of interest statement

The authors declare that the research was conducted in the absence of any commercial or financial relationships that could be construed as a potential conflict of interest.
